# Medical Students' Awareness of Smell Loss as a Predictor for Coronavirus Disease 2019

**DOI:** 10.3389/fpubh.2020.597897

**Published:** 2020-12-09

**Authors:** Turki Aldrees, Sharif Almatrafi, Turki Aldriweesh, Mohammad Mokhatrish, Abdulaziz Salamh, Feras Alkholaiwi

**Affiliations:** ^1^Department of Otolaryngology-Head and Neck Surgery College of Medicine, Prince Sattam Bin Abdulaziz University, Al-Kharj, Saudi Arabia; ^2^Department of Otolaryngology-Head and Neck Surgery College of Medicine, Imam Mohammad Ibn Saud Islamic University, Riyadh, Saudi Arabia

**Keywords:** COVID-19, smell loss, coronavirus, anosmia, medical student

## Abstract

**Background:** Anosmia has been reported as an early presentation of coronavirus disease 2019 (COVID-19). However, the pathophysiological mechanism of olfactory dysfunction is still unclear.

**Aim:** The aim of this study to evaluate the knowledge regarding common symptoms, anosmia, treatment options, and PPE among medical students in three different universities of Saudi Arabia.

**Methods:** This cross-sectional survey conducted among medical students in Saudi Arabia. Google Forms was used to create the survey. The questionnaire included demographic information, knowledge of COVID-19 symptoms, sources of information, and the level of awareness of specific symptoms (loss of smell and taste).

**Results:** A total of 494 students completed the questionnaire. The majority of the participants were aware of common COVID-19 symptoms like fever and cough (79.8 and 67.2%, respectively), but less than half were aware that smell or taste dysfunction might be a symptom of COVID-19 (44.3 and 30.2%, respectively). The present study revealed that the source of information also plays a critical role in medical students' awareness regarding the symptoms of COVID-19. Students using international organization's websites, medical databases, or published research had better knowledge of anosmia as a COVID-19 symptom compared to those who used WhatsApp, Google, or unofficial social media pages. In our study, a minority (11.9%) of the participants relied on unofficial social media pages as the main source of their information.

**Conclusion:** Saudi medical students understand that smell or taste dysfunction can be a potential symptom of COVID-19, but this knowledge was not as widespread as the knowledge regarding the most common COVID-19 symptoms.

## Introduction

Near the end of 2019, cases of atypical pneumonia of unknown etiology were discovered in Wuhan in the mainland of China. Later on, these cases proved to be linked to a viral infection caused by a novel virus of the coronavirus family; the International Committee on Taxonomy of Viruses had named it severe acute respiratory syndrome coronavirus 2 (SARS-CoV-2) (1). The infection spread, leading to a major outbreak around the world. In February 2020, the World Health Organization (WHO) labeled this disease an epidemic and designated it coronavirus disease 2019 (COVID-19) (2). As the disease spread, multiple outbreaks were reported, leading to WHO announcing this disease to be a worldwide pandemic in early March 2020. In Saudi Arabia, the first positive case of COVID-19 was reported on March 2 in a Saudi citizen who had traveled to Iran through Bahrain (3).

The victims of this disease mostly presented with fever, cough, and shortness of breath (4, 5). Furthermore, smell and taste dysfunction have also been reported as a symptom of this new disease, which can affect patient quality of life (6–8).

Although the pathophysiological mechanism of olfactory dysfunction is still unclear, in many studies, anosmia has been reported as an early presentation of COVID-19. In one study, anosmia was the only presenting nasal symptom in 94% of confirmed patients (9). Similarly, in a recent systematic review, it was found that 41% of COVID-19 confirmed patients had olfactory dysfunction (10). Patients with a combination of anosmia and flu-like symptoms have been reported to be up to 10 times more likely to test positive for COVID-19 than if this combination is not present (11, 12). Consequently, many medical associations and societies have added this symptom to a list of screening tools for COVID-19 (13, 14).

As this epidemic continues to cross borders, it is imperative that medical providers be aware of prevention and early detection methods for this disease. Smell and taste dysfunction have been neglected symptoms, and the awareness of these symptoms needs to be emphasized. In this paper, we aim to determine the knowledge regarding common COVID-19 symptoms, smell and taste dysfunction symptoms and available treatment options/outcome, using personal protective equipment (PPE), and the primary source of those information, among medical students in Saudi Arabia.

## Methods

This cross-sectional survey was conducted among medical students in Saudi Arabia from June to July 2020. The survey was created in Google Forms and distributed via email and WhatsApp through medical student representatives and vice deans of medical colleges. The questionnaire was developed by the authors based on recent available literature. A pilot study was conducted to determine the clarity of developed questionnaire. The questionnaire included students' demographic information, current college year, university affiliation, knowledge of common symptoms of COVID-19 (fever and cough), and knowledge of potential changes to or loss of smell or taste caused by COVID-19. Furthermore, students were surveyed about their level of awareness of available treatment options for loss of smell based on the recent literature. In addition, students were asked about their sources of primary information regarding COVID-19. A five-point Likert scale ranging from strongly disagree to strongly agree was used to record participants' responses. The questionnaire took an average of 11 min to complete.

Sample size was based on Raosoft's sample size calculator. The latest available estimate for the number of medical students enrolled in Saudi universities was 26,216 students. A sample size of 379 was chosen based on a 5% margin error and 95% confidence interval. However, a larger sample size of 494 students was obtained with a respond rate of 61%. A standardized general description about the study objective was included in the survey, and students voluntarily answered the questions.

The statistical analyses were performed in SPSS 26. Frequencies, descriptive statistics, normality tests, the *t*-test, the Mann–Whitney *U*-test, one-way analysis of variance (ANOVA), the Kruskal–Wallis test, correlation, and the chi-square test of independence were computed.

Four indicators were calculated based on the questionnaire. The common symptoms knowledge index was comprised of Q7 and Q8, the anosmia/dysgeusia knowledge index was Q9–Q18, the personal protective equipment (PPE) knowledge index was Q19–Q23, and the treatment option knowledge index was Q24–Q28. The response scale ranged from 1 (strongly agree) to 5 (strongly disagree), with the exception of Q16, which ranged from 1 (within 2 weeks) to 5 (I don't know). The answers were coded as 0 (incorrect) and 1 (correct) and summed up for each index.

In the next step, the anosmia/dysgeusia index, PPE index, and treatment index were summed up to create a triple index. Then, both the common symptoms index and triple index were recalculated into percentages of correct answers. Based on percentages, a category was assigned: poor knowledge (0% for common symptoms, ≤60% for triple index), moderate (50% for common symptoms, 61–80% for triple index), and good (100% for common symptoms, >80% for triple index).

## Results

Table 1 shows the cohort characteristics. A total of 494 students completed the survey, and 319 (64.6%) were male. Additionally, 95 students (19.2%) were in their fourth year, and King Khalid and King Saud University accounted for the largest proportion of participants.

**Table 1:** Demographic profile of the sample.

**Variable/Group**	***n* (%)**
**Gender**	
Female	175 (35.4%)
Male	319 (64.6%)
**Age**	
M (SD)	22.14 (2.41)
**College year**	
First year	56 (11.3%)
Second year	72 (14.6%)
Third year	62 (12.6%)
Fourth year	95 (19.2%)
Fifth year	79 (16.0%)
Sixth year	48 (9.7%)
Internship	82 (16.6%)
**University affiliation**	
King Saud University	86 (17.4%)
Prince Sattam Bin Abdulaziz	83 (16.8%)
Imam Muhammad ibn Saudy	37 (7.5%)
King Saud bin Abdulaziz University for Health Sciences	62 (12.6%)
Al Qassim University	56 (11.3%)
King Khalid University	87 (17.6%)
King Abdulaziz	61 (12.3%)
Other	22 (4.5%)
**Information sources**	
Whatsapp	5 (1.0%)
Unofficial social media	59 (11.9%)
Official governmental accounts	288 (58.3%)
Google News	16 (3.2%)
Medical databases/published research	43 (8.7%)
TV	10 (2.0%)
International organization sites (e.g., WHO)	73 (14.8%)
**COVID knowledge level**	
Complete knowledge	153 (31.0%)
Partial knowledge	294 (59.5%)
Little knowledge	45 (9.1%)
No knowledge	2 (0.4%)

Table 2 shows the frequencies and percentages of responses of the included participants regarding the knowledge of COVID-19. The majority of participants 427 (52.8%) knew that rise in body temperature as the symptom of corona disease. Likewise, 44.9% knew that cough, 60.1% knew that shortness of breath, and 35.6% knew that complete loss of sense of smell as the major symptom of corona disease. Furthermore, 36.3% of participants did not know that change in taste or taste of eating as the symptom of the corona. 48.7% of participants did not agree that loss or change of sense of smell requires hospital admission. Similarly, 51.7% of participants did not know that loss or change of sense of smell does not interfere with hospitalization. 35.7% did not know that sudden loss or change of sense of smell as the only symptom of corona infection. Moreover, 319 participants (39.4%) responded to question (In average, how long does the sense of smell lose last from COVID19) “within 20 weeks,” while 49 (6.1%) chose “1 month.” Twenty-eight respondents (3.5%) answered with “more than 1 month.” The majority did not know the answer (413, 51.1%).

**Table 2:** Frequencies and percentages of answers.

	**Strongly agree**	**Agree**	**Don't know**	**Disagree**	**Strongly disagree**
**Common symptoms knowledge index**					
Q7. COVID 19 Symptom of Fever	394 (79.8%)	98 (19.8%)	0 (0.0%)	0 (0.0%)	2 (0.4%)
Q8. COVID 19 Symptom of Cough	332 (67.2%)	132 (26.7%)	10 (2.0%)	18 (3.6%)	2 (0.4%)
**Anosmia/dysgeusia knowledge index**					
Q9. COVID 19 Symptom of Smell loss	219 (44.3%)	158 (32.0%)	99 (20.0%)	16 (3.2%)	2 (0.4%)
Q10. COVID 19 Symptom of Taste Changes	149 (30.2%)	159 (32.2%)	138 (27.9%)	40 (8.1%)	8 (1.6%)
Q11. COVID 19 Symptom of Smell Change Frequently Associate with Nasal Obstruction	58 (11.7%)	144 (29.1%)	224 (45.3%)	56 (11.3%)	12 (2.4%)
Q12. COVID 19 Symptom of Loss of Smell Commonly Associate with Sever Covid19 Cases	24 (4.9%)	74 (15.0%)	213 (43.1%)	154 (31.2%)	29 (5.9%)
Q13. Symptom of Loss of Smell Has been added as Criteria for COVID 19 Diagnosis	103 (20.9%)	140 (28.3%)	223 (45.1%)	23 (4.7%)	5 (1.0%)
Q14. Sudden Loss of Smell Mandate Isolation	134 (27.1%)	148 (30.0%)	150 (30.4%)	53 (10.7%)	9 (1.8%)
Q15. Any Patient with Isolate Sudden of Smell Loss Recommended to be Screen for COVID19	166 (33.6%)	186 (37.7%)	103 (20.9%)	36 (7.3%)	3 (0.6%)
Q17. Majority of Patient recovered their smell	153 (31.0%)	171 (34.6%)	156 (31.6%)	12 (2.4%)	2 (0.4%)
Q18. Majority of Patient complains of smell loss after been diagnosed with COVID19	20 (4.0%)	84 (17.0%)	234 (47.4%)	131 (26.5%)	20 (4.0%)
**Personal protective equipment knowledge index**					
Q19. Wearing N95 Mask During Contact with Patients with Sudden Smell Loss Secondary to COVID19	272 (55.1%)	103 (20.9%)	85 (17.2%)	26 (5.3%)	8 (1.6%)
Q20. Wearing Face Shield During Close Contact Examination	244 (49.4%)	93 (18.8%)	111 (22.5%)	33 (6.7%)	13 (2.6%)
Q21. Wearing Body Gown During Close Contact	242 (49.0%)	91 (18.4%)	102 (20.6%)	37 (7.5%)	22 (4.5%)
Q22. Wearing Shoes Cover During Close Contact	183 (37.0%)	92 (18.6%)	134 (27.1%)	50 (10.1%)	35 (7.1%)
Q23. Wearing Head Cover During Close Contact	215 (43.5%)	91 (18.4%)	117 (23.7%)	46 (9.3%)	25 (5.1%)
**Treatment option knowledge index**					
Q24. Smell Training Programme is Recommended for Smell Loss	88 (17.8%)	81 (16.4%)	289 (58.5%)	35 (7.1%)	1 (0.2%)
Q25. Nasal Steroid Spray is Recommended for Smell Loss	65 (13.2%)	100 (20.2%)	283 (57.3%)	35 (7.1%)	11 (2.2%)
Q26. Oral Steroid Spray is Recommended for Smell Loss	33 (6.7%)	46 (9.3%)	328 (66.4%)	67 (13.6%)	20 (4.0%)
Q27. Omega Supplement is Recommended	31 (6.3%)	51 (10.3%)	351 (71.1%)	48 (9.7%)	13 (2.6%)
Q28. Using Vitamin A Nasal Drops is Recommended	43 (8.7%)	65 (13.2%)	348 (70.4%)	31 (6.3%)	7 (1.4%)

### Group Differences

Table 3 demonstrates the results of the Mann-Whitney *U*-test to highlight the differences. The findings on difference in knowledge regarding COVID-19 symptoms, anosmia, PPE, and treatment based on gender is shown. It was found that, both male and female had similar knowledge on symptoms, anosmia and treatment options. However, the knowledge of PPE is significantly higher in female participants as compared to the male participants.

**Table 3:** Gender comparison using *t*-test/Mann-Whitney *U*-test.

	**Female**			**Male**			***t/U***	***p***
	**M**	**SD**	**Me**	**M**	**SD**	**Me**		
Common symptoms	1.94	0.23	2.00	1.93	0.27	2.00	−0.49	0.622
Anosmia	5.02	2.28	5.00	5.08	2.36	5.00	0.24	0.812
PPE	3.67	1.85	5.00	3.08	1.97	3.00	23437.50	0.002
Treatment	1.09	1.35	0.00	1.32	1.46	1.00	25571.50	0.101

### College Year

No significant differences were found for any of the four knowledge indices in terms of college year (*p* = 0.692 for common symptoms index, *p* = 0.733 for the anosmia index, *p* = 0.742 for the PPE index, and *p* = 0.220 for the treatment index).

### University

Two noted some differences, but that they were not significant were identified in terms of the anosmia index. Namely, students from Al Qassim University (M = 4.39, SD = 2.43, Me = 5.00) scored significantly lower than students from Al Imam Muhammad Ibn Saud Islamic University Imam Muhammad ibn Saud University (M = 5.84, SD = 2.30, Me = 6.00; p =0.086) and students from other universities (M = 6.09, SD = 1.87, Me = 6.00; *p* = 0.094). Table 4 showed university names cross different indexes.

**Table 4:** Frequencies for symptoms and triple index categorization in universities.

	**Common symptoms index**			**Triple index**		
	**Poor**	**mod**.	**Good**	**Poor**	**mod**.	**Good**
King Saud University	1 (1.2%)	8 (9.3%)	77 (89.5%)	65 (75.6%)	21 (24.4%)	0 (0%)
Prince Sattam Bin Abdulaziz University	0 (0%)	4 (4.8%)	79 (95.2%)	60 (72.3%)	17 (20.5%)	6 (7.2%)
Imam Muhammad ibn Saud University	0 (0%)	3 (8.1%)	34 (91.9%)	22 (59.5%)	11 (29.7%)	4 (10.8%)
King Saud bin Abdulaziz University for Health Sciences	0 (0%)	4 (6.5%)	58 (93.5%)	42 (67.7%)	17 (27.4%)	3 (4.8%)
Al Qassim University	0 (0%)	0 (0%)	56 (100%)	46 (82.1%)	10 (17.9%)	0 (0%)
King Khalid University	0 (0%)	8 (9.2%)	79 (90.8%)	66 (75.9%)	20 (23.0%)	1 (1.1%)
King Abdulaziz University	0 (0%)	3 (4.9%)	58 (95.1%)	50 (82.0%)	9 (14.8%)	2 (3.3%)
Other	0 (0%)	0 (0%)	22 (100%)	15 (68.2%)	6 (27.3%)	1 (4.5%)

### Information Sources

In terms of anosmia knowledge, students who got their information from international organization's websites (M = 5.96, SD = 2.02, Me = 7.00) scored significantly higher than those who used WhatsApp (M = 2.20, SD = 2.28, Me = 2.00; *p* = 0.007), Google News (M = 3.56, SD = 2.99, Me = 2.00; *p* = 0.003), unofficial social media pages (M = 4.61, SD = 2.13, Me = 5.00; *p* = 0.015), and official government accounts (M = 4.93, SD = 2.18, Me = 5.00; *p* = 0.012). A similar pattern was found for users of medical databases and published research (M = 6.00, SD = 2.88, Me = 7.00), who exhibited significantly higher anosmia knowledge than those who used WhatsApp (M = 2.20, SD = 2.28, Me = 2.00; *p* = 0.009), Google News (M = 3.56, SD = 2.99, Me = 2.00; *p* = 0.005) and unofficial social media pages (M = 4.61, SD = 2.13, Me = 5.00; *p* = 0.047). There was also a some noted non-significant difference in anosmia knowledge between users of medical databases (M = 6.00, SD = 2.88, Me = 7.00) and users of official government accounts (M = 4.93, SD = 2.18, Me = 5.00; *p* = 0.082).

With regard to the PPE index, students who used Google News (M = 1.69, SD = 1.49, Me = 1.00) as an information source scored significantly lower than students who used medical databases (M = 3.49, SD = 1.91, Me = 4.00; *p* = 0.03), international organization's websites (M = 3.79, SD = 1.72, Me = 5.00; *p* = 0.002), and official government accounts (M = 3.29, SD = 1.96, Me = 4.00; *p* = 0.025). No significant differences were found for the treatment index.

### Knowledge Level

Respondents who assessed their knowledge of COVID-19 as low (M = 1.80, SD = 0.40, Me = 2.00) exhibited significantly lower knowledge of common symptoms compared to students who stated that they had complete (M = 1.95, SD = 0.22, Me = 2.00; *p* = 0.001) and partial (M = 1.96, SD = 0.21, Me = 2.00; *p* < 0.001) knowledge. Students who claimed to have a complete level of knowledge on COVID-19 (M = 6.03, SD = 2.17, Me = 6.00) exhibited significantly higher knowledge of anosmia than participants with partial (M = 4.71, SD = 2.23, Me = 5.00; *p* < 0.001) and little (M = 4.13, SD = 2.50, Me = 5.00; *p* < 0.001) knowledge.

A similar pattern was found for the PPE index. Participants with a self-assessed complete level of COVID-19 knowledge (M = 3.72, SD = 1.80, Me = 5.00) scored significantly higher than those with little (M = 2.51, SD = 2.24, Me = 2.00; *p* = 0.004) and partial (M = 3.20, SD = 1.93, Me = 4.00; *p* = 0.024) knowledge. Finally, students who self-assessed as having complete knowledge of COVID-19 (M = 1.46, SD = 1.48, Me = 1.00) exhibited significantly higher knowledge of treatment in comparison to participants with self-assessed partial knowledge (M = 1.11, SD = 1.38, Me = 0.00; *p* = 0.041).

## Discussion

The current study aimed to determine the knowledge regarding COVID-19 common symptoms, anosmia (loss of sense of smell and taste), treatment options, and PPE among the medical students from three different universities. The findings revealed that majority of the participants had significant knowledge related to COVID-19 general symptom such as rise in body temperature, cough, and shortness of breath. However, only 35.6% participants were aware of anosmia (loss of sense of smell) as a specific symptom of corona disease. A viral infection is the most common cause of anosmia. Like other viruses, coronaviruses can result in anosmia in 10–15% of patients (14). COVID-19 infection differs from other coronaviruses in that the chemosensory dysfunction is more prevalent and is not associated with other rhinitis symptoms like nasal obstruction and rhinorrhea (11). There are growing evidence reported that loss of taste and smell is a strong predictor of COVID-19 infection (12). Having said that, in this study a 36.3% of participants did not know that change in taste or taste of eating as the symptom of the corona. One study reported that out of 6,452 confirmed COVID-19 cases, 64.76% experienced anosmia or ageusia (15). A possible pathogenesis for anosmia suggested by Brann et al. (16) is that the COVID-19 virus affects the non-neuronal olfactory cells, causing loss of smell and associated taste alteration. Zhou et al. (17) confirmed that COVID-19 uses the cellular angiotensin-converting enzyme 2 receptor. Because this enzyme is distributed in the oral cavity, it is possible that the virus affects the taste function (17).

In terms of gender differences regarding the knowledge of symptoms, anosmia, treatment, and PPE. The findings revealed that female participants had significantly good knowledge (*p* = 0.020) on the use of PPE as compared to that male participants. However, there was no significant difference in the knowledge level of symptoms, anosmia and treatment between male and female participants. It contradicts the findings of a previously conducted study which states that showed that men had less knowledge of COVID-19 compared to women (18).

Overall, our study revealed that medical students in Saudi Arabia have good knowledge of common COVID-19 symptoms, regardless of their information source. The same finding was seen in two cross-sectional studies conducted in Jordan and Uganda, where the students showed a high level of knowledge (19, 20). The current study also revealed that no significant difference was seen when the knowledge was assessed based on college year or university affiliation. These findings might be explained by the awareness campaigns and education programs conducted by governments to target the whole community (18). Another factor that may explain these findings is the seriousness of the disease, especially after being declared a pandemic by the WHO (21).

Our findings show that the majority of the included participants were aware of common COVID-19 symptoms like fever and cough (79.8 and 67.2%, respectively), but less than half were aware that smell or taste dysfunction might be a symptom of COVID-19 (44.3 and 30.2%, respectively). Because anosmia can be an early symptom of COVID-19, this lack of knowledge may delay the diagnosis of COVID-19 and thus increase the risk of infection spread (22).

In India, the health ministry proposed provisional permission of senior medical undergraduates to treat patients with COVID-19 (23). Medical students in the university hospitals are at risk of infectious diseases. Proximity to infected patients' respiratory droplets increases the risk of disease transmission (24). It has been shown that cooperation between hospitals and universities enhances medical students' knowledge of new infectious diseases and helps to improve their perceptions and preventive behaviors (25). Therefore, it is important to assess their knowledge of COVID-19 symptoms and preventive measures. To the best of our knowledge, this is the first descriptive study in this field among undergraduate medical students from all regions of Saudi Arabia.

The assessment of smell or taste dysfunction is not only helpful for diagnosing COVID-19 but also for triaging patients. In a previous study of 417 mild to moderate COVID-19 patients, 85.6 and 88.0% of patients reported olfactory and gustatory dysfunctions, respectively. Moreover, olfactory dysfunction appeared before the other symptoms in 11.8% of cases (22). Moein et al. (26) suggested that early quantitative smell testing, like the University of Pennsylvania Smell Identification Test, may help to identify COVID-19 patients in need of early treatment or quarantine. Interestingly, in our study, 150 participants (30.4%) were not aware that a sudden change in the sense of smell necessitates isolation and seeking medical help, and 62 participants (12.5%) disagreed with this statement.

The present study revealed that the source of information is also critical to the awareness of COVID-19 symptoms among medical students. We found that students using international organization's websites, medical databases, or published research had better knowledge of anosmia as a COVID-19 symptom compared to those who used WhatsApp, Google News, or unofficial social media pages. The Ugandan cross-sectional study mentioned above also found that although the majority of the students used mass media to obtain their information, those who also used journal articles and websites had significantly greater knowledge than the others (20).

There is widespread dissemination of misinformation in social media websites (27). However, in our study, a majority of participants relied on authentic governmental sources such as WHO and ministry of health websites for information regarding COVID-19. This was contradictory to the Jordanian and Ugandan studies, which revealed that social media was the main source of the students' information (19, 20). Although the CDC has recommended PPE for healthcare workers when dealing with suspected or confirmed COVID-19 patients (28), our study revealed that the students had a suboptimal level of PPE knowledge overall. We also found that participants who self-assessed as having a complete level of COVID-19 knowledge scored significantly higher than those with little or partial knowledge regarding the PPE knowledge index.

In their systematic review, Costa et al. (7) stated that there is still no scientific evidence of specific treatments for olfactory and taste disorders in COVID-19. They also showed that recovery generally occurs within the first 2 weeks after the resolution of COVID-19. Our study revealed that the majority of the participants were unaware of the treatment options described in the literature, and only 210 participants (42.5%) knew that recovery usually occurs within 2 weeks of resolution of the disease.

There are certain limitations to this study. Firstly, the study design was cross-sectional, which provides lower quality. However, a longitudinal study was not a feasible option with the current situation of social distancing and lockdown. The second limitation was convenience sampling. There is a potential risk of bias as the underprivileged populations may be ignored due to the lack of access to the social media platforms such as Whatsapp and Twitter. The findings of the current study are limited as it may not be the actual representative of the Saudi population. It can be addressed in the future studies through more systematic sampling method that ensures representativeness and generalization of the findings. The third limitation in the current study is related to study instrument. It has not been rigorously validated for the potential factors that influence knowledge, perceptions and attitudes of the participants. Some of the potential factors that might influence knowledge include health literacy and risk perception. Moreover, one another limitation was the possibility of participants to give false responses. It has been seen that in self-reported data the participants strive to give socially desirable responses. Therefore, the data may not be the true representation of the knowledge, attitude and practices regarding the COVID-19 symptoms.

## Conclusion

The present study revealed good knowledge of COVID-19 symptoms among medical students in Saudi Arabia. Additionally, it was found that Saudi medical students understand that smell or taste dysfunction can be a potential symptom of COVID-19, but this knowledge was not as widespread as the knowledge regarding the most common COVID-19 symptoms, such as fever and cough. Universities need to enhance the knowledge of COVID-19 among medical students and prepare them for safe practice once needed.

## Data Availability Statement

The raw data supporting the conclusions of this article will be made available by the authors, without undue reservation.

## Ethics Statement

The studies involving human participants were reviewed and approved by Prince Sattam bin Abdulaziz University. The patients/participants provided their written informed consent to participate in this study.

## Author Contributions

All authors listed have made a substantial, direct and intellectual contribution to the work, and approved it for publication.

## Conflict of Interest

The authors declare that the research was conducted in the absence of any commercial or financial relationships that could be construed as a potential conflict of interest.
